# Investigating Ideomotor Cognition with Motorvisual Priming Paradigms: Key Findings, Methodological Challenges, and Future Directions

**DOI:** 10.3389/fpsyg.2012.00519

**Published:** 2012-11-23

**Authors:** Roland Thomaschke

**Affiliations:** ^1^Institut für Psychologie, Universität RegensburgRegensburg, Germany

**Keywords:** motorvisual priming, dual task, ideomotor theory, binding, planning and control model, action-effect blindness, categorical perception

## Abstract

Ideomotor theory claims that perceptual representations of action-effects are functionally involved in the planning of actions. Strong evidence for this claim comes from a phenomenon called motorvisual priming. Motorvisual priming refers to the finding that action planning directly affects perception, and that the effects are selective for stimuli that share features with the planned action. Motorvisual priming studies have provided detailed insights into the processing of perceptual representations in action planning. One important finding is that such representations in action planning have a categorical format, whereas metric representations are not anticipated in planning. Further essential findings regard the processing mechanisms and the time course of ideomotor cognition. Perceptual representations of action-effects are first activated by action planning and then bound into a compound representation of the action plan. This compound representation is stabilized throughout the course of the action by the shielding of all involved representations from other cognitive processes. Despite a rapid growth in the number of motorvisual priming studies in the current literature, there are still many aspects of ideomotor cognition which have not yet been investigated. These aspects include the scope of ideomotor processing with regard to action types and stimulus types, as well as the exact nature of the binding and shielding mechanisms involved.

## Introduction

Human behavior is to a large degree anticipative and goal-directed. That means most of our actions are not merely direct responses to environmental stimuli, but are selected with regard to an anticipated action goal. How anticipated action goals are cognitively processed in action selection is an extensively researched area in cognitive psychology (e.g., Nikolaev et al., 2008; Nattkemper et al., 2010; Pfister et al., 2012). Currently one of the most influential theories in this area is the ideomotor theory (Massen and Prinz, 2009; Shin et al., 2010). The fundamental claim of ideomotor theory is that anticipated action goals processed in action selection are represented as the *sensory* consequences of achieving those goals. To put it another way, action selection involves perceptual representations of action-effects (Kunde et al., 2007; Waszak et al., 2012). Various versions of ideomotor theory have emerged in the cognitive psychology literature during the last three decades (see Kunde et al., 2007; Nattkemper et al., 2010; Shin et al., 2010, for reviews). Despite some conceptual differences between these versions, all variations are based on two essential hypotheses: first, goal-directed behavior is achieved by goal representations which have a functional role in action selection. Second, the goal representations are represented in the same format as sensory input from these goal states would be represented (Prinz, 1997).

Although the ideomotor theory has a long history in philosophy and psychology (Stock and Stock, 2004; Pfister and Janczyk, 2012), it has evolved with increasing rapidity only since the late 1990s, owing to a growing number of empirical findings supporting the involvement of perception in action processing (see Nattkemper et al., 2010; Shin et al., 2010, for reviews). During this time a set of classical ideomotor paradigms has emerged.

One example is the response-effect-compatibility paradigm (Kunde, 2001, 2003, 2004; Koch and Kunde, 2002; Rieger, 2007; Janczyk et al., 2009; Pfister et al., 2010). In response-effect-compatibility experiments, participants provide free or forced choice responses, which have task-irrelevant effects. Effects can be compatible (i.e., naturally following on from the current response, e.g., a left stimulus following a left key press), or incompatible. Responses are on average faster when they are followed by compatible effects than by incompatible ones. A performance decrement when action and effect are constantly mismatched indicates that response processing is sensitive to action-effect matching, and involves, thus, some representations of effects (Hoffmann et al., 2001).

Another classical paradigm in ideomotor research is the effect-learning paradigm (Elsner and Hommel, 2001, 2004; Hommel et al., 2003; Kray et al., 2006; Hoffmann et al., 2009). The logic is similar to the response-effect-compatibility design, the only difference being that the action-effect associations are acquired only during the experiment, in an initial learning phase. In a seminal study by Elsner and Hommel (2001) participants pressed two keys in an arbitrary self-chosen sequence. The keys were contingently followed by tone effects. After that, either a forced or a free choice test phase was employed (differing between experiments and studies). In a forced choice test phase, the former action-effects now figured as action cues. Participants were faster when the cue response mapping matched the cue effect mapping experienced in the learning phase than when the mapping was reversed (see also Herwig et al., 2007; Herwig and Waszak, 2009). In a free choice test phase, where the former action-effects figured as Go-signals, participants chose more often than chance would suggest the response which had been followed, in the learning phase, by the current Go-signal (see also Hoffmann et al., 2009; Pfister et al., 2011).

Further prominent paradigms in the context of the ideomotor theory include versions of the Serial Response Time paradigm (Nissen and Bullemer, 1987) that emphasize the formation of R-S associations (Ziessler, 1998; Ziessler and Nattkemper, 2001), and studies where human movement stimuli induce compatible response tendencies in observers (Knuf et al., 2001; De Maeght and Prinz, 2004; Prinz et al., 2005; Häberle et al., 2008; Watanabe, 2008).

Although research with these paradigms has produced significant knowledge about ideomotor mechanisms, they provide only relatively indirect access to the processing of perceptual representations in action. In these paradigms, the process of action selection can only be primed *in advance* by perceptual activation. The effectiveness of perceptual effect-like primes on the consecutive action is interpreted as evidence for the involvement of perceptual representations in the selection of these actions. A more direct experimental access to ideomotor cognition would require measuring perceptual processing online, *during* action planning. This strategy is realized in motorvisual priming paradigms.

### Motorvisual priming

**Figure 1:**
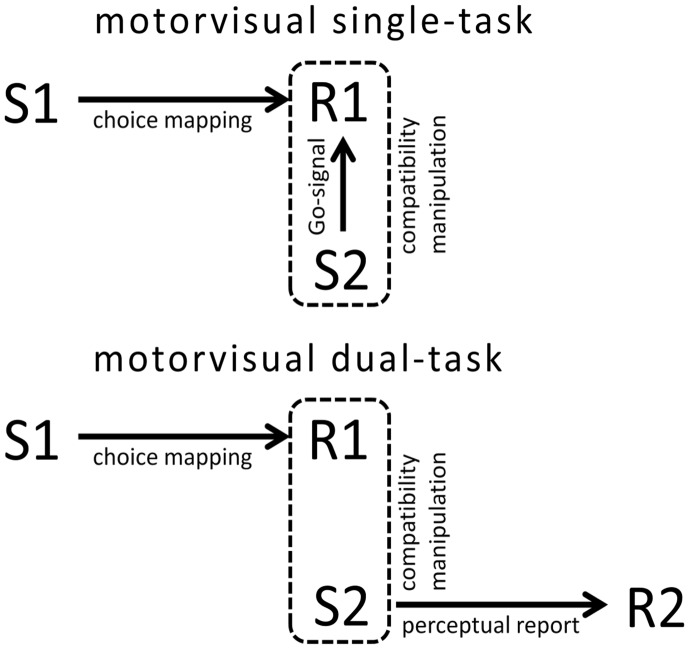
**Schematic illustration of motorvisual single tasks and motorvisual dual tasks**. In both types of tasks R1 is selected according to a perceptual cue S1. During R1 preparation, a target stimulus S2 is presented. The experimenter manipulates whether R1 and S2 are ideomotor-compatible or not, that is, whether S2 resembles, in any respect, an effect of R1. In single tasks S2 figures as Go-stimulus for R1. In dual tasks S2 identity is reported by a secondary response R2. Thus, R1 and S2 are functionally unrelated. A motorvisual priming effect is present when R1-S2 compatibility affects R1 performance in single tasks, or when R1-S2 compatibility affects R2 performance in dual tasks.

In motorvisual priming paradigms, a response action (R1) is selected and executed in response to a perceptual cue (S1), while, concurrently, a stimulus (S2) has to be detected or identified (see Figure 1). The experimenter manipulates whether S2 is ideomotor-compatible with R1 (i.e., whether on any dimension it is similar to an effect of R1) or not. This compatibility usually affects the speed or accuracy of S2 perception. Such compatibility effects are commonly seen as originating from an involvement of perceptual representations of effect-compatible stimuli in action planning (Kunde and Wühr, 2004).

Motorvisual priming paradigms can, on the one hand, be realized as *single* task versions (Craighero et al., 2002). In this case, S2 mostly figures as a Go-Signal for R1 (see Figure 1). The identity of R1 is cued by S1, but R1 is withheld until presentation of S2. Although the identity of the Go-Signal is irrelevant to the task, its ideomotor-compatibility with the withheld response still has an effect on the response correctness and latency (Craighero et al., 1999). These effects are commonly interpreted as reflecting the processing of perceptual representations in action planning (Craighero et al., 2002; Bortoletto et al., 2011).

On the other hand, motorvisual priming paradigms have also been realized in *dual task* versions. In these studies R1 is planned according to S1 and executed either immediately (Müsseler and Wühr, 2002; Wühr and Müsseler, 2002), at the participant’s own pace (Eder and Klauer, 2007; Oriet et al., 2007), or after a compatibility-neutral Go-Signal (Kunde and Wühr, 2004; Hommel and Müsseler, 2006). At different times during the preparation or execution of R1, a second stimulus S2 is displayed. S2, in contrast to single task versions, is not a Go-Signal for R1 here, but belongs to a second unrelated task instead (see Figure 1). The second task consists in reporting the identity of S2 by a secondary – either speeded (Zwickel et al., 2007; Pfister et al., 2012) or unspeeded (Müsseler and Hommel, 1997a; Stevanovski et al., 2002) – response R2. Although R1 and S2 belong to different tasks and are functionally unrelated, influences of ideomotor-compatibility between R1 and S2 on R2 performance have frequently been observed. These effects are commonly interpreted as being owed to the involvement of perceptual representations in R1 processing. In R1-S2 compatible trials, this involvement interferes with S2 perception, and this interference is reflected by R2 performance (Müsseler, 1999; Eder and Klauer, 2009).

Motorvisual priming is, of course, not the only way in which actions can affect perception of ideomotor-compatible stimuli. There is accumulative evidence that motor-expertise with certain movement types (e.g., athletics or dancing) can selectively improve the perception of movements of this type (Calvo-Merino et al., 2005; Craig et al., 2009; Hohmann et al., 2011; Cañal-Bruland et al., 2012; Diersch et al., 2012). A similar long-term motorvisual effect has been observed in experimentally controlled motorvisual learning studies. Motorvisual learning experiments typically include a motor-learning phase, where the participants acquire new movement patterns, and a visual test phase, where the participants have to observe similar movement patterns. Results typically show that movement practice selectively improves or biases perceptual capacities for stimuli similar to the motor-practiced ones (Hecht et al., 2001; Casile and Giese, 2006; Engel et al., 2008a,b; Beets et al., 2010; Glenberg et al., 2010). Both learning phenomena can be interpreted as evidence for the involvement of perceptual representations in motor execution (Vogt and Thomaschke, 2007).

Although such motorvisual learning transfer studies are of eminent importance in determining visuomotor processing in skill acquisition, they are, however, of limited value for the detailed investigation of ideomotor mechanisms. As in the aforementioned effect-learning paradigms, inferences are drawn from relations between different experimental phases. These conclusions are informative about how one acquires action-effect associations, but are less informative about the mechanisms by which the acquired action-effects bring about intended actions. For that reason, motorvisual priming studies pose a much more powerful and temporally precise tool, because these paradigms allow manipulation and measurement of ideomotor processes in an online, trialwise fashion. Consequently, the present review focused on motorvisual priming studies, although occasional evidence from learning studies is cited when directly related to the findings from priming studies.

### Motorvisual priming and ideomotor theory

Motorvisual priming studies have frequently been cited as support for the ideomotor theory (Stoet and Hommel, 1999; Kunde and Wühr, 2004; Shin et al., 2010). Actually, only rarely has any other explanation been offered for motorvisual priming than the processing of perceptual representations in action (see, however, Oades and Kreul, 2001, for an exception). Indeed it seems difficult to explain why actions should affect unrelated perceptual events, if perceptual representations are *not* involved in action processing. Thus, motorvisual priming can be regarded as one of the most convincing cases of empirical evidence for ideomotor theory’s central claim that action planning processes perceptual action-effects. The reference to motorvisual priming in the ideomotor literature is quite general, however. It usually does not go beyond citing motorvisual priming as support for the theory in general. This is in stark contrast with the high informative value that motorvisual priming studies have for the understanding of many aspects of processing perceptual effect representations in action selection.

The aim of the present review is to show that previous motorvisual priming studies allow precise conclusions about the detailed functional role that perceptual representations play in action planning. Furthermore I discuss potential methodological pitfalls in designing motorvisual priming studies, and sketch directions for future motorvisual priming research which might further elucidate the mechanisms of ideomotor cognition.

## How are Perceptual Effect Representations Processed in Action Planning?

In the following two subsections, I review evidence from motorvisual priming studies on different aspects of ideomotor cognition. First, it will be argued that results from motorvisual priming studies allow the nature of perceptual representations processed in action planning to be determined, and that the findings are in accordance with predictions of current theories of ideomotor cognition. Second, motorvisual evidence for the binding of perceptual action-effects in action plans is discussed. Motorvisual priming studies have shown that action selection activates and binds effect representations throughout planning and execution, in order to stabilize the action plan against interferences.

### Ideomotor cognition processes categorical representations of action-effects

As reviewed above there is plenty of evidence for the involvement of perceptual representations in action processing. One important question which has not been answered in the action-effect learning studies previously discussed is the format which these perceptual representations have. Are they perceptual representations of a categorical symbolic nature or are they metric spatial representations? In this subsection I will show that motorvisual priming studies can answer this question, and that it is in favor of categorical representations.

There is accumulative interdisciplinary evidence that human cognitive processing makes use of two fundamentally different kinds of mental representations (Kosslyn, 1987, 2006; Logan, 1995; Kosslyn et al., 1998; Okubo et al., 2010). One class of mental representations is commonly referred to as *categorical*. Categorical representations are of a relational nature. They are used to represent cognitive entities – like stimuli or responses – as members of categories. Typical examples include word identities, affective categories (positive/negative), or abstract propositional spatial categories (e.g., above/below, or left/right).

Categorical representations are usually defined in opposition to *metric* representations. Metric representations are coordinate representations of exact spatial relations. Examples include the coordinate location of a stimulus or an effector, or the exact size or rotational angle of an object (Kosslyn, 1994).

#### Categorical representations in planning and control

In order to answer the question of which representations are subject to ideomotor cognition, one must take into account that action processing is commonly conceived of as consisting of two different sub-processes, planning and control, and that these sub-processes differ with respect to the processed representations.

Most theories of motor cognition distinguish between movement planning processes and movement control processes (Elliott et al., 2001; Glover et al., 2012). Planning processes integrate motivational, environmental, and goal-anticipative factors in order to determine the appropriate action in a given situation. Action planning does not specify in advance the entire course of the movement until its completion, but instead determines only the gross parameters in order to initiate the movement (e.g., which effector, which initial direction, etc., see Schmidt, 1975; Hommel, 2005, 2010).

Movement control, on the other hand, comes into play once the movement is chosen and initiated. Movement control specifies the exact movement parameters online via fast feedback cycles. Control constantly compares predictions based on the actual state of the movement with incoming sensory information, in order to minimize mismatch between movement goal and prospective course of the movement (Wolpert et al., 1998; Wolpert and Ghahramani, 2000; Bubic et al., 2010). Movement control works at high speed and can rapidly correct for movement disturbances and perturbations in target size, location, or orientation (Prablanc and Pélisson, 1990; Gosselin-Kessiby et al., 2008; Hesse and Franz, 2009; van de Kamp et al., 2009).

Glover (Glover, 2002, 2004; Glover and Dixon, 2002) has suggested that movement planning primarily processes categorical representations whereas control processes only metric information. This proposal has been supported by a considerable amount of evidence from empirical studies that compared the effects of categorical and/or metric manipulations on planning and control processes (see Glover, 2004; Thomaschke et al., 2012a, for reviews). For example, Keulen et al. (2004) have shown that priming a reaching task with the gross layout of the target distracter distribution, pertinent to categorical coding, affected only the early planning stage of grasping. The locational coordinate distance between distracter and target, likely to infer with metric processing, on the other hand, affected only later control stages of the grasping movements. In a dual task study, Liu et al. (2008) showed that a letter identification task affected a secondary pointing movement task only in RT (a measure of planning duration), but not in accuracy (a measure of control effectiveness). Likewise, Spiegel et al. (2012) found that planning a grasping movement impairs a secondary verbal working memory task, but grasping control does not. Hesse and Deubel (2011) showed that a digit identification task affected initial movement parameters in the early phase of a grasping movement, whereas the later, control-based, phase of the movement was unaffected. See Glover and Dixon (2002) and Thomaschke et al. (2012b) for further evidence that planning processes categorical information and control processes metric information.

These findings lead to a clear conclusion concerning the type of perceptual representations in ideomotor cognition. As the ideomotor principle – that action selection involves perceptual effect processing – concerns action selection, not action control, ideomotor theory is commonly assumed to relate exclusively to the action planning stage, not to action control (Kunde et al., 2007; Shin et al., 2010; Janczyk et al., 2012). Consequently, the ideomotor theory would suggest that the perceptual representations processed in motor cognition are of a categorical nature.

#### Types of mental representation in motorvisual priming

Motorvisual priming studies provide a simple straightforward test for the ideomotor theory’s prediction that perceptual representations in action planning are categorical. When motorvisual priming effects are owed to perceptual processing in action selection, then one would assume that such effects can only be observed for dual tasks where the overlap between R1 and S2 is categorical rather than metric.

At first glance, the empirical findings are clearly at odds with this prediction. Motorvisual priming has been observed for categorical R1-S2 overlap (e.g., Kunde and Wühr, 2004), but also for instances where the overlap can unambiguously be described as metric (e.g., sharing a certain spatial location, see Deubel et al., 1998), and for stimuli where it is ambiguous whether the overlap is categorical or metric (e.g., biological stimuli, Jacobs and Shiffrar, 2005; Miall et al., 2006).

Closer inspection reveals, however, that the effect direction in motorvisual priming research systematically varies between different studies, depending on the type of the representations involved. Some have reported motorvisual impairment, whereas perception was facilitated by compatible action in other studies (Schütz-Bosbach and Prinz, 2007; see Muthukumaraswamy and Johnson, 2007; Press et al., 2009; Thomaschke et al., 2012a; and Zwickel and Prinz, 2012, for systematic discussions of this issue). The effect direction allows a clear distinction to be drawn between motorvisual effects owed to action planning and motorvisual effects owed to other action-related processes.

One important aspect of action planning processes is that they integrate and stabilize the features of a selected movement. This ensures that the basic features of the movement (e.g., which effector is moved) remain constant throughout its course. This, however, requires that movement features are shielded against access by other cognitive processes, including other action alternatives, including perceptual processes (Müsseler, 1999; Stoet and Hommel, 1999). As perceptual effect representations, according to ideomotor theory, are also features of an action plan, these features are also shielded against other cognitive processes, including perception. Thus, ideomotor theory would predict that action planning impairs the perception of effect-compatible stimuli, instead of facilitating it (Hommel et al., 2001; Hommel, 2009).

##### Types of mental representation in motorvisual interference

When we consider only motorvisual impairment studies among the motorvisual priming studies – that is, studies where the motorvisual priming effect can unambiguously be ascribed to perceptual processing in action planning – the overlap between R1 and S2 is exclusively on a categorical dimension. Motorvisual impairment has been shown for speaking and identifying direction words (Hommel and Müsseler, 2006), or color words (Kunde and Wühr, 2004), and for writing and identifying letters of certain forms (James and Gauthier, 2009). Other examples include impairment from left/right key presses on left/right pointing arrow symbols (Müsseler and Hommel, 1997b), and impairment from positively/negatively charged lever movements on the detection of positive/negative words (Eder and Klauer, 2007, 2009). Hence, motorvisual priming studies have confirmed the ideomotor theory’s prediction that ideomotor cognition relates exclusively to categorical representations (see also Zwickel et al., 2010a, for a discussion of this issue).

Some authors have divided categorical representations further into *verbal* categorical representations and *spatial* categorical representations (Kemmerer and Tranel, 2000; Tranel and Kemmerer, 2004; van der Ham and Postma, 2010; van der Ham and Borst, 2011). A motorvisual priming study by Hommel and Müsseler (2006), has shown that both kinds of categorical representations can figure as perceptual effect representations in action selection. In one experiment speaking the words left/right impaired the identification of the written words left/right in compatible trials compared with incompatible trials (Exp. 3B). The overlap in this experiment can clearly be regarded as categorical verbal. In an analogous experiment (Exp. 1A), with left/right key presses as R1, and left/right pointing arrow symbols as S2, the overlap was categorical spatial. Again, a motorvisual impairment effect was observed. In further experiments with the cross-conditions, spoken words with arrow heads (Exp. 2B), and key presses with written words (Exp. 3A), no motorvisual impairment effect was observed. The absence of an effect in the latter experiments confirmed that two different kinds of categorical representations were applied. Thus, action selection involves spatial and verbal categorical representations.

Further support for the strict confinement of ideomotor cognition to categorical representations comes from the finding that motorvisual priming effects – that are not related to action selection (see above) – have been observed *only* for metric spatial R1-S2 overlap on non-categorical dimensions. These studies can be classified in motorvisual priming studies with non-biological metric R1-S2 overlap, and with biological motion stimuli. I discuss each type in turn.

##### Motorvisual facilitation with metric representations

When R1 and S2 overlap on a metric dimension, effects are either absent (Bonfiglioli et al., 2002) or in a positive direction (Hommel and Schneider, 2002; e.g., Paprotta et al., 1999; Wykowska et al., 2009, 2011, 2012) in the sense of better performance when R1 and S2 are metrically compatible than when they are not. Motorvisual facilitation effects are thought to reflect action control, instead of action selection (Hommel, 2009, 2010; Thomaschke et al., 2012a). Action control is a closed loop process leading to rapid constant online updating of precise metric information in order to correct potential target movement mismatches (Wolpert et al., 1998; Wolpert and Ghahramani, 2000). This process benefits greatly from attentional pre-selection of movement-relevant areas in perceptual space. Thus actions, once selected, strongly facilitate processing of compatible metric representations like effector laterality (Hommel and Schneider, 2002; Koch et al., 2003; Müsseler et al., 2005; Press et al., 2009; Gherri and Eimer, 2010), goal location (Fischer, 1997; Linnell et al., 2005), orientation (Lindemann and Bekkering, 2009; Janczyk et al., 2012), or size (Fagioli et al., 2007a,b; Symes et al., 2008, 2010). This motorvisual facilitation effect is, in contrast to action planning, independent from action context (Fischer et al., 2007; Thomaschke et al., 2012b) but it is selective with regard to the specific control demands of different movement types (Fischer and Hoellen, 2004). For example, pointing movements particularly facilitate locational metric processing (Deubel and Schneider, 2004; Collins et al., 2008), whereas grasping facilitates metric size processing (Symes et al., 2008) and orientation processing (Lindemann and Bekkering, 2009; see Memelink and Hommel, 2012, for a review of motorvisual facilitation with different movement types).

Two recent works have directly compared motorvisual facilitation and impairment in one and the same study, and have confirmed that impairment is obtained with categorical R1-S2 overlap and facilitation can be observed with metric R1-S2 overlap. Koch (2009) found a motorvisual dual task facilitation effect in response times with a metric visual task (orientation judgment) and motorvisual interference with an analogous categorical visual task (object naming). Thomaschke et al. (2012b) found that left key presses impaired perception of symbols pointing to the left (categorical overlap), but facilitated stimulus perception in the left visual field (metric overlap).

Thus, evidence from motorvisual priming studies with metric R1-S2 overlap fully confirms the prediction of ideomotor theory. As metric representations are not involved in ideomotor cognition, these paradigms have not yielded motorvisual impairment effects (as would have been characteristic of perceptual effects from action selection), but have exclusively produced motorvisual facilitation effects.

At this point, two clarifications are needed to clearly distinguish between the processes related to action selection and those related to action control. One concerns feedback loop processing in motor cognition, and the other concerns categorical activation by relative location.

*Closed loops in action planning*. Note that some versions of the ideomotor theory also assume a kind of a feedback loop (see Nattkemper et al., 2010; Hughes et al., 2012; Waszak et al., 2012, for reviews). In particular, it is assumed that the action selection process is shaped by information about whether actions have successfully achieved their intended action-effect or not. Therefore perceptual representations of action-effects can be associated more precisely with appropriate generative actions, which, in turn, makes action selection more effective (Ziessler et al., 2004). These feedback loops do, however, relate rather to the acquisition of action-effect associations, instead of the actual online processing of perceptual representation in action-perception. Consequently, they are not likely to affect perception during action selection.

*Categorical activation by relative location*. Responses and stimuli always have metric representations of location, simply because stimuli and responses necessarily occupy a location in physical space. When metrical information is important for response control, processing of metrically compatible stimuli is facilitated (e.g., in the left visual field, for left pointing movements, see above). Depending on task context, however, the location of a stimulus can also activate a categorical, relational representation of its position, which is employed in action planning. When, for example, a stimulus frequently appears in different locations which are relative left/right to each other, it automatically activates categorical left/right representations, even when the stimulus location is task-irrelevant. Such effects have been observed in Simon effect (Proctor and Vu, 2006) studies, and in response-effect-compatibility studies (see introduction) with response compatible locations. Kunde (2001), for example has shown that horizontally arranged finger press responses to non-spatial imperative stimuli are facilitated more when the responses are followed by locationally compatible visual stimuli than when they are followed by incompatible stimuli (see above). In this study, response planning has activated categorical representations of response location, and categorical representations of effect location, which have been compatible in one condition and incompatible in the other. Consequently, in the former condition, response planning was facilitated. Note that the results from motorvisual facilitation studies reviewed here strongly suggest that (owing to the metric overlap and control relevance) effect perception was facilitated in the compatible condition relative to the incompatible one. This was, however, not measured in Kunde’s study.

##### Motorvisual facilitation for human movement stimuli

There is accumulative behavioral and neuroscientific evidence that the rapid metric visuomotor feedback loops employed in action control also have a function in the perception and prediction of others’ movements. Behavioral (Catmur and Heyes, 2011; Heyes, 2011; Martel et al., 2011; Boyer et al., 2012) and neuroscientific (Saygin et al., 2004) studies have shown that the observation of others’ movements covertly activates the own motor system in a compatible way (see also the examples of ideomotor induction in section Introduction). The covert motor activation is likely to launch the same movement control processes as an active movement would have done (Wilson and Knoblich, 2005; Fagioli et al., 2007a). These control processes constantly produce predictions for the next immediately expected perceptual input caused by the movement, based on the current state of the motor system (Wolpert et al., 1995). For actually executed movements, such predictions have the function of detecting and correcting for mismatches between action goal and predicted course of the action (see above). Wilson and Knoblich (2005) have recently argued that these predictions are also employed in the observation of others’ actions. They could serve to stabilize the ongoing percept by assisting perceptual disambiguation (Wilson and Knoblich, 2005). This visual function of motor control is reflected in motorvisual facilitation effects in dual tasks which apply biological motion displays (Miall et al., 2006). In particular, metric positional prediction of future visual movement states is facilitated when compatible movements are planned or executed (Graf et al., 2007; Springer et al., 2011, 2012; Saygin and Stadler, 2012; Stadler et al., 2012).

As this perceptual function of action is not dependent on action planning, however, and is thus not in the domain of ideomotor cognition, ideomotor theory would predict no motorvisual interference effects with biological stimuli. The data from motorvisual priming studies are in line with this prediction. The effects are mostly facilitative. Only when the temporal asynchrony between executed and observed movement is too extreme for predictions to be perceptually supportive have interferences been observed (see Christensen et al., 2011, for a review).

#### Conclusion

Evidence from motorvisual priming studies shows that ideomotor cognition is confined to categorical representations. Motorvisual priming has been shown for almost all kinds of representations. Effect direction, however, allows the motorvisual effect caused by ideomotor processes to be identified, because these processes typically lead to motorvisual impairment. Motorvisual impairment has only been observed with categorical stimuli. Motorvisual facilitation effects, on the other hand, have only been shown with metric representations and with biological stimuli. These effects are owed to motor control processes, and are, consequently, not in the domain of ideomotor theory.

### Action plans bind active perceptual representations during action execution

Motorvisual priming paradigms are informative, not only about the nature of perceptual representation in action planning, but also about the way in which these representations are processed. The duration of motorvisual priming effects suggests that perceptual representations are bound in action plans to shield them from competing processes. Furthermore, the boundary conditions for motorvisual priming to occur suggest that action planning first activates perceptual representations before binding them. I discuss each issue in turn.

#### The duration of motorvisual interference

In early studies on motorvisual impairment priming, the effect was explained in terms of refractoriness of perceptual representation by action planning (Müsseler and Hommel, 1997a). These explanations assumed that perceptual representations are briefly activated during action selection, just at the point when they are employed to inform motor parameter choice in an ideomotor fashion. According to these explanations, the impaired availability of the action-effect representation for concurrent perceptual processes results from refractory inhibition of the representation following its brief ideomotor activation. Hence, the reduced availability of action-effect representations for other processes would have been only a byproduct of ideomotor cognition, without own functional value. This account of motorvisual impairment suggests a rather narrow time window for the effect, near the time of action execution (see Wühr and Müsseler, 2001, for a discussion).

Contrary to this prediction, however, in further investigations of motorvisual impairment, the effect has been observed during a relatively long time window, spanning from at least 2000 ms before action execution (Wühr and Müsseler, 2001, Exp. 2) until 1000 ms after action execution (Müsseler and Wühr, 2002; Stevanovski et al., 2002, Exp. 1; Oriet et al., 2003a,b; Wühr and Müsseler, 2002).

These findings have led to the interpretation of the motorvisual impairment effect as an indicator of something more essential in ideomotor cognition than a byproduct caused by refractoriness. Stoet and Hommel (1999) have suggested that action selection entails binding processes which connect all selection-relevant features of an action into a common event file (Hommel, 2004). Perceptual representations of action-effects are also features of an action and are, according to ideomotor theory, selection-relevant. Thus, these representations are also bound into event files. These binding mechanisms stabilize action plans through the course of their execution, and therefore shield the action plan against interferences from other cognitive processes, like, for example, other competing action plans. They can also prevent the same action being cyclically triggered again and again by the activated effect anticipations (Müsseler, 1999). Since the mid-2000s, a considerable amount of evidence has been accumulated in favor of event file binding in action planning (see, e.g., Colzato et al., 2006; Hommel, 2007; Mattson and Fournier, 2008; Wiediger and Fournier, 2008). Binding of features into action plans has also been referred to as “occupation” (Schubö et al., 2004) or “encapsulation” (Müsseler, 1999). Based on the prolonged time course of motorvisual interference, Wühr and Müsseler (2001) have concluded that motorvisual impairment is caused by the binding of perceptual event representations in compound representations of the action plan. This view has now become common sense in motorvisual interference research (Hommel, 2004; Nishimura and Yokosawa, 2010a; Thomaschke et al., 2012b).

#### The boundaries of binding

Stoet and Hommel (1999) suggest that action selection consists of two phases. First, the relevant action features are activated, and after activation they are quickly bound into a composite representation of the action. The binding of activated action features remains intact throughout the course of the movement. Motorvisual priming studies have supported this view with regard to perceptual representations of action-effects. Particularly informative are studies on situations where the second phase of Stoet and Hommel’s model – the binding phase – had either not yet commenced, was already over, or was prevented by certain task demands. I will discuss each of these issues in turn.

##### Motorvisual priming before binding

According to Stoet and Hommel, action features are first activated and then bound. In order to investigate the transition between both phases directly one would have to measure motorvisual priming effects at the point when features are activated but not yet bound. In the majority of motorvisual interference studies, this condition is, however, not met. Usually R1 is executed at leisure after S1, and S2 perception is measured shortly before, shortly after, or during execution of R1 (e.g., Müsseler et al., 2000, 2001; Eder and Klauer, 2007; Nishimura and Yokosawa, 2010a). Under such a scenario, R1 features can be assumed to be long activated and bound when participants initiate the movement and S2 is presented.

A study by Müsseler and Wühr (2002) has, however, applied speeded R1 responses and has measured S2 perception almost immediately after S1. At this point it can be assumed that the S2 compatible perceptual representations are already activated by R1 selection but not yet bound. Müsseler and Wühr (2002, Exp. 2) applied a relatively difficult speeded four-choice task with intervals of 200, 400, or 1000 ms between S1 and S2. They observed the typical impairment effect at 400 and 1000 ms, but a motorvisual facilitation effect was observed at 200 ms. Participants needed on average around 600 ms for their speeded responses to the cues. This indicates that S1-R1 translation was particularly difficult in this task and binding followed activation after more than 200 ms.

##### Motorvisual priming after binding

Other studies have investigated motorvisual priming *after* the binding phase. When S2 is presented at increasing time intervals after R1 execution, the motorvisual interference effect gets significantly weaker (Oriet et al., 2003b, Exp. 1, 2007; Wühr and Müsseler, 2001, Exp. 1). In some studies the priming effect turned into facilitation at the longest interval. For example, Müsseler et al., 2001, Exp. 2) had three timing conditions. R1 was to be executed immediately after a self-paced neutral double key press. S2 was presented at the double key press, at R1, or 500 ms after R1. The typical motorvisual impairment effect was found at the former conditions. When S2 was presented 500 ms after R1, however, a motorvisual facilitation effect was observed (see Schubö et al., 2004, for a similar pattern).

These results can be seen as further support for the two-phase view of action planning. After action execution, binding is not required any longer and consequently released, but activation in the action features, including perceptual representations of action-effects, still persists, and consequently causes motorvisual facilitation, when S2 is presented late after R1 (see also James and Gauthier, 2009, for a related discussion).

##### Motorvisual priming without binding

Another important source of information concerning the activation/binding view of action planning is motorvisual priming studies with movement tasks that counteract the binding process. A study by Caessens and Vandierendonck (2002) has been particularly illuminating in this respect. They applied a Stop-Signal paradigm, where participants had to execute speeded lateral key presses as R1 in response to visual S1. In half of the trials, a stop-signal appeared 200 ms after S1. In the latter case participants had to refrain from executing R1. After a variable SOA, a masked arrowhead was presented as S2. In one experiment (Exp. 1A), the typical motorvisual impairment from R1 planning on the perception of compatible S2 was observed. In a further experiment (Exp. 1B), however, Caessens and Vandierendonck increased the difficulty of the Stop-Signal procedure. Again, in half of the trials, a stop-signal was presented but the interval between S1 and the stop-signal was individually adapted by a staircase procedure such that participants were only able to refrain from responding in half of the Stop-Signal trials. Thus, binding of the response features into a composite representation in order to shield them from other processes would have been counterproductive here. In half of the trials this action plan would have had to be abandoned in favor of a new plan to inhibit the prepared action. Release of action features would have taken time, hindering quick inhibition. Under these experimental conditions, a motorvisual facilitation effect was observed, reflecting feature activation, but not binding.

This finding suggests that binding only takes place when stabilization of a chosen action is of advantage. In situations with high action uncertainty, where action plans need to be quickly abandoned and rapidly replanned very often, action features are activated by ideomotor processes, but not bound.

#### Conclusion

Motorvisual priming studies have provided conclusive evidence about the processing of perceptual representations in action planning. When perceptual representations are employed to select actions in an ideomotor fashion, these representations are first activated, to the effect that compatible perceptual processes are facilitated. Then these representations are quickly bound, together with other action features, into a composite action representation, shielding them from involvement in other cognitive processes. The binding process is only abandoned in situations where one has to switch quickly between opposing action options.

## Methodological Considerations

Despite the importance of motorvisual priming paradigms for investigating ideomotor processes, there is an inherent methodological difficulty in measuring such effects which requires careful consideration and control. Most behavioral cognitive psychology paradigms are visuomotor paradigms in a very general sense. The experimenter systematically manipulates the participant’s perceptual stimulation as an independent variable and records the participant’s responses. This basic logic of psychological experiments is designed to test hypotheses about causal effects from stimulus presentation on response production. Working in this intuitive way, stimulus manipulation and response measurement are thought to reveal regularities in mental processing from perception to action. Stimuli are perfectly controllable and directly affect perceptual processing, whereas responses are typically caused by internal mental processes. This experimental design appears intuitively feasible since it meets our everyday experiences with perceptions and actions. Perceptual stimulation is experienced as being largely caused by the environment. We usually have to change the environment (e.g., shifting objects into our visual field) to influence perceptual stimulation (yet, it has sometimes been argued that a scientific description of perception should not follow this intuition, e.g., Gibson, 1979; Noë, 2004; Bompas and O’Regan, 2006). Actions, on the contrary, are experienced as being produced or at least largely shaped by our own cognitive system. Motorvisual priming experiments have to reverse this highly intuitive causal direction (just as ideomotor theory does on a conceptual level). Such experiments aim at establishing a causal effect of response execution on stimulus perception. In order to do this, an experimenter would have to directly control the action intentions of the participants as an independent variable and directly measure the content or other features of their visual perception as a dependent variable. Both are practically impossible. Although one can induce involuntary movements by neural stimulation or by applying external forces to effectors, voluntary action planning (often of central interest in motorvisual research and constituting the central explanatory goal of ideomotor theory) cannot be directly physically controlled by the experimenter in a way comparable with stimulus manipulation in visuomotor experiments. Likewise visual perception is an event inside the participant’s brain, which cannot directly be observed, and neuroscientific measurements are not precise enough to differentiate between perceptual states to a degree that could reasonably be assumed to be affected by action. Hence, motorvisual researchers have to apply indirect methods of response manipulation and indirect measures of visual perception. Both can lead to characteristic methodological problems, as will be discussed in turn.

In the remainder of this section, I discuss potential alternative non-motorvisual explanations for motorvisual priming studies arising from those methodological problems. I also show how these potential confounds have been dealt with in previous studies.

### Transitivity of stimulus similarity

The indirect manipulation of participants’ action planning processes, as independent variable, is usually achieved by varying experimental instructions. In some paradigms, the instruction to prepare a certain type of action is blocked. In order to avoid learning effects, however, most motorvisual priming paradigms vary the response randomly from trial to trial. This is commonly done by displaying a response cue before each trial. The cue signals the response required in the current trial. In some trials the cued response is compatible with the observed visual stimulus, in others it is incompatible. A motorvisual interaction is detected by comparing visual performance for compatible and incompatible response-stimulus pairings. The compatibility relation between stimulus and response is usually a very natural one and is a salient feature of each (e.g., matching gestures, words, movement directions, or common spatial locations). The instructed mapping between cue and response, however, is also often a natural and intuitive one. This ensures that the cue response translation does not absorb too much cognitive capacity by requiring participants to memorize and apply complex rules, which could lead to a deficit in response correctness.

These requirements, to keep both the instructed cue response mapping and the evaluated response-stimulus compatibility relation simple and intuitive, makes it tempting to choose similar or even identical compatibility mappings for both. Doing so, however, leads to serious problems concerning the interpretation of a potential compatibility interaction, because in such situations compatibility between response and stimulus is always accompanied by compatibility between response cue and stimulus. When compatibility between cue and response and between response and stimulus are defined in the same terms, then any systematic compatibility effect of response-preparation on stimulus perception is indistinguishable from a compatibility effect of the cue on stimulus perception (see also Hommel and Müsseler, 2006, for a discussion of this issue).

Consequently, studies that apply analogous compatibility definitions for the cue response mappings and for response-stimulus matching cannot be regarded as unambiguous evidence of a motorvisual effect. Any compatibility effect could be owed to a causal response-preparation stimulus perception link as well as to a causal cue-perception stimulus perception link (the latter being a visuovisual interaction). The motorvisual priming literature has however suggested several strategies to control for this potential interpretation problem.

For example, Müsseler and Hommel (1997a, Exp. 1, 2), Müsseler and Hommel (1997b, Exp. 1, 2) used the same stimuli (arrow heads) for S1 cues and for S2 stimuli with identical cue response and response-stimulus compatibility definitions. The effect was also found, however, in motorvisual impairment experiments that applied more complex cue response mapping. Müsseler and Hommel cued the response with direction words instead of arrows (Müsseler and Hommel, 1997a, Exp. 4) and reversed the natural cue response mapping from the original experiment (Müsseler and Hommel, 1997a, Exp. 5), whereas Müsseler et al. used auditory cues (Müsseler et al., 2000, Exp. 1) and required the participants to generate responses endogenously in an alternating sequence (Exp. 2).

These findings show that one of the most extensively researched motorvisual priming paradigms (i.e., the priming of arrow perception by lateral key presses) cannot be explained by visuovisual effects.

### Transitivity of response similarity

A comparable interpretation problem arises from the necessity to measure stimulus perception indirectly in motorvisual experiments. Perceptual performance is usually assessed by involving a secondary response in the design. The secondary response R2 is either a speeded detection/identification of the stimulus (e.g., Craighero et al., 2002; Pfister et al., 2012) or an unspeeded report of certain stimulus features (e.g., Müsseler and Hommel, 1997a) or a reproduction of the stimulus movement (Schubö et al., 2004). The speed or accuracy of R2 is a measure of the speed or accuracy of the perceptual process. As regards S1-R1 mapping, however, there are arguments for keeping the S2-R2 mapping relatively natural and intuitive. This is especially important for *speeded* secondary responses. A complex translation would be likely to require additional cognitive processing time and thereby add an additional source of variance to the response time, which would interfere with the statistical detection of any response-stimulus compatibility effects. Yet, when R1-S2 compatibility and S2-R2 compatibility are defined by the same mapping rules, the compatibilities cannot vary independently of each other. In such a situation a compatibility priming effect could not be assigned unambiguously to motorvisual priming since it would be indistinguishable from a primary-response secondary-response priming effect. Response–response priming effects have frequently been observed in dual tasks with compatibility relations between functionally unrelated responses (Schuch and Koch, 2004; Wenke and Frensch, 2005). This interpretability problem can also be controlled for, however. For example, Müsseler and Hommel (1997a, Exp. 1), Müsseler and Hommel (1997b, Exp. 1) used the same key pressing movements as primary and secondary response with the same compatibility definition but they also obtained a motorvisual interference effect when, in a control experiment, the secondary responses were verbal responses (direction words) instead of key presses (Müsseler and Hommel, 1997a, Exp. 2).

An analogous criticism applies to Schubö et al. (2001, 2004) motorvisual interference paradigm. The secondary response in their paradigm figures as primary-response in the subsequent trial. Thus, the compatibility mapping between response and stimulus is identical with the mapping between stimulus and secondary response. Schubö et al. (2004, Exp. 2) attempted to rule out a response secondary response explanation by including an additional motor task (drawing circles) between trials. They found comparable compatibility effects with and without such a task. According to their interpretation, the motor task would have interfered with, and thus eliminated, a response secondary response compatibility effect.

### Visuomotor explanations in motorvisual priming experiments

As reviewed in the introduction, visual processing can directly affect motor processing, evidenced by influences of task-irrelevant aspects of visual stimulation on motor action. When stimuli and responses are compatible, responses are faster and more accurate than with incompatible ones. Some of these visuomotor effects have been interpreted as evidence for the ideomotor theory. When the compatibility relation between stimulus and response is an action-effect relation – i.e., when response performance is better when responses are triggered by their typical perceptual effects than when they are triggered by non-effects – such findings can clearly be attributed to ideomotor processing, because they show that perceptual effect representations play a role in action selection.

There is, however, also plenty of evidence for visuomotor priming where the relation between stimulus and response is not one of effect but one of affordance. In such instances, the stimulus is not a typical effect of the action, but usually rather precedes the action in the sense of affording it. For example, the task-irrelevant side of a handle on a cup primes the ipsilateral response hand (Fischer and Dahl, 2007; Bub and Masson, 2010; Goslin et al., 2012). These kinds of visuomotor priming effects can also be explained by associative learning accounts (Heyes, 2001) instead of ideomotor theory, without assuming any perceptual processing in action selection. In some visuomotor priming studies it is fully apparent, whether the compatibility between stimulus and response rests on the stimulus typically being an external imperative cause of the response (affordance priming), or whether it rests on the stimulus typically being an external effect of the response (ideomotor priming).

For many other visuomotor studies, it is, however, unclear whether the relation between stimulus and response is one of affordance or one of effect. This has led to controversies about the appropriate interpretation of visuomotor effects with affordance/effect-ambiguous stimulus-response pairs.

For example, it has been debated whether visuomotor priming for biological motion stimuli, sometimes referred to as “imitation priming,” is owed to associative learning (Heyes, 2001, 2003; Heyes and Ray, 2004; Bird and Heyes, 2005; Heyes et al., 2005; Wiggett et al., 2011) or to ideomotor principles (Brass et al., 2000; Stürmer et al., 2000), because in imitation a compatible stimulus can be an affordance cue from the perspective of the imitator *and* an effect from the perspective of the model (see, however, Leighton et al., 2010, for an integrative view). A similar interpretation ambiguity pertains for the Simon effect – a priming effect from irrelevant stimulus laterality on ipsilateral responses (Proctor and Vu, 2006). On the one hand, actions are often afforded by ipsilateral stimuli (Michaels and Stins, 1997), but, on the other hand, they equally often have ipsilateral effects (Greenwald and Shulman, 1973).

This issue is of particular importance for the interpretation of motorvisual priming paradigms, because for many types of S2 stimuli commonly applied in these paradigms, it is not apparent whether they are compatible with R1 in an affordance sense or in an effect sense. If, however, the designer of a motorvisual experiment with affordance/effect-ambiguous stimuli can make sure that the experiment really demonstrates an influence of action processing on perceptual processing, then this effect can definitely be ascribed to ideomotor processing, despite the ambiguity of the stimuli. The just described alternative non-ideomotor explanations for visuomotor priming with affordance/effect-ambiguous stimuli do not apply to motorvisual paradigms. These non-ideomotor accounts can easily explain why perceptions that usually trigger certain responses prime these responses, but they cannot explain why these responses should prime perceptions which usually trigger them. Thus, motorvisual paradigms are, for theoretical reasons, superior to visuomotor paradigms with regard to the investigation of ideomotor processing with rather ambiguous stimuli. This is an important advantage, because there are few stimuli which can be classified without doubt as effect, and not as affordance, of a response, unless they are associated with the response in a pre-experimental learning phase (as, e.g., in Cardoso-Leite et al., 2010; Pfister et al., 2012).

As mentioned above, however, this advantage is only realized when the experimental design of a motorvisual priming study does not allow an alternative visuomotor explanation. For some motorvisual priming studies this is not the case. When these studies apply affordance/effect-ambiguous stimuli, they cannot be definitively regarded as informative about ideomotor processing. This applies in particular to motorvisual single task paradigms and to concurrent motorvisual dual task paradigms. I will discuss each in turn.

#### Single tasks

For both affordance/effect-ambiguous stimulus classes discussed above (lateral stimuli and human movement stimuli), single tasks have been interpreted as evidence for motorvisual effects. Van der Lubbe and colleagues (Van der Lubbe and Abrahamse, 2011; Van der Lubbe et al., 2012), for example, have suggested a framework that explains the standard Simon effect in terms of motorvisual effects (see also Metzker and Dreisbach, 2009, 2011; Nishimura and Yokosawa, 2010b), and Stürmer et al. (2000) and Craighero et al. (2002) have interpreted imitation priming in terms of the ideomotor theory.

Craighero et al. (2002), for instance, primed stimulus perception by the preparation of compatible or incompatible grasping movements. The secondary response was the speeded execution of the previously prepared movement. They explained the effect as the effects of motor preparation on stimulus perception. The effect could also be interpreted, however, as an effect of stimulus perception on response execution, as Grosjean and Mordkoff (2001), Vogt et al. (2003) and Miall et al. (2006) have pointed out.

A strategy to avoid this interpretation ambiguity has been applied by Lindemann and Bekkering (2009). They investigated motorvisual effects by a series of single tasks, and protected the effects against visuomotor explanations with an additional motorvisual dual task.

#### Concurrent dual tasks

An alternative visuomotor explanation for motorvisual dual tasks is only possible when stimulus and response are cyclic, temporally extended, events (e.g., Hamilton et al., 2004; Schubö et al., 2004; Jacobs and Shiffrar, 2005; Miall et al., 2006; Zwickel et al., 2010b). From now on, I will refer to such tasks by the term *concurrent* motorvisual task.

Concurrent motorvisual priming effects are behaviorally indistinguishable from visuomotor effects. Several previous studies have shown that it is more difficult to perform compatible cyclic movements in synchrony with compatible stimulation than incompatible stimulation (Kilner et al., 2003; Bouquet et al., 2011; Capa et al., 2011; Press, 2011; Gowen and Poliakoff, 2012). This means that the difficulty of the motor task differs between compatible and incompatible trials in concurrent motorvisual priming studies. In compatible trials, the motor task is more difficult. Performing a more demanding task might lead to an *unspecific* impairment of general perceptual performance in incompatible trials. Unspecific means that the impairment is *per se* independent of the action’s compatibility with the perceptual event, but would affect perception of any stimulus (see Müsseler and Wühr, 2002, for an analysis of specific and unspecific motorvisual interference). Unspecific motorvisual priming effects have often been demonstrated in dual tasks, where R1-S2 compatibility was either not manipulated or additive to unspecific impairment (Band et al., 2006; Johnston and McCann, 2006; Brisson and Jolicœur, 2007). Unspecific motorvisual impairment can, however, not be regarded as clear evidence for ideomotor processing. It can also be explained by limitations in either motor- or perceptually-related processes alone, such as transfer of information to visual short-term memory (Jolicœur and Dell’Acqua, 1998), or response selection (Pashler, 1994; Marois et al., 2006), owing to limited general processing capacities. Motorvisual evidence for the ideomotor theory requires that actions impair perception in a content-sensitive, compatibility-selective, manner, because only this shows that specific perceptual effect representations are processed in action planning.

The best strategy to ensure that a motorvisual priming effect can be explained by compatibility-specific motorvisual impairment, instead of a combination of compatibility-specific visuomotor impairment and unspecific motorvisual impairment, would be to have the S2 stimulus temporally follow the R1 response (e.g., Oriet et al., 2003b).

### Conclusion

Although motorvisual priming studies are a powerful tool for investigating perceptual processing in motor cognition, they are sometimes susceptible to alternative explanations. This explanation ambiguity stems from the requirement to manipulate responses indirectly as independent variables, and to measure perceptual processes indirectly as the dependent variable. Alternative explanations can be excluded however, by using dual tasks, where response and stimulus do not temporally overlap, and where S1-R1 mapping is defined on another dimension as R1-S2 compatibility.

## Directions of Future Research

Although previous motorvisual priming studies have substantially extended our knowledge about ideomotor processing, many questions about perceptual processing in action planning are still unanswered, and there is enormous potential for future motorvisual priming research. In the following subsections I sketch some of the most urgent ideomotor issues that could be solved by motorvisual priming research.

### The function of binding

Motorvisual priming research has shown that perceptual features are bound into action plans, and are, consequently, not fully accessible to concurrent perceptual processes. The function of this binding process is, however, not clear yet. Some have suggested that binding of the perceptual effect representations keeps these representations from triggering the same action redundantly again and again by ideomotor mechanisms. In that case, execution would be blocked by a repetitive chain of triggering the same action (e.g., Müsseler, 1999). According to this account, the function of effect-binding would be the inhibition of outgoing activation from the perceptual effect representations toward other motor processes. Thus, the perceptual impairment would be merely a perceptual side-effect of inhibiting representations to shield them from actions.

Koch and Prinz (2002) suggested an account of effect-binding, which presents motorvisual impairment not as a side-effect but as the main function of binding. They say that “… the code subserving response execution is shielded against interference from visual input, which then leads to an impairment in perceiving compatible stimuli” (Koch and Prinz, 2002, p. 200). According to this view, R1 production is shielded against any interference from irrelevant visual information which might affect it. S2 is task-irrelevant for R1 production, but would be a potential ideomotor-trigger in R1-S2 compatible trials. Thus, shielding is particularly important in compatible trials and would produce the motorvisual impairment effect.

There is preliminary evidence for both accounts. The finding that binding can also affect compatible *responses* in dual tasks (e.g., Mattson and Fournier, 2008; Eder et al., 2012), rather supports the proposal that the function of binding is to avoid redundant repetitive response planning.

Support for the shielding account comes from studies on the modulation of shielding processes. According to Dreisbach (2012) the process of shielding responses against interference from irrelevant stimuli does depend strongly on the task set applied, that is, on how the response is cued. When it is a simple arbitrary S-R mapping, such shielding is virtually absent, whereas a constant (Dreisbach and Wenke, 2011) and rule-based (Dreisbach and Haider, 2008, 2009) mapping leads to substantial shielding effects.

This pattern is indeed also reflected in motorvisual impairment. Thomaschke et al. (2012b) and Wühr and Müsseler (2002) investigated the role of different S1-R1 mapping rules on a motorvisual impairment effect. Both studies compared motorvisual (R1-S2) impairment effects under compatible S1-R1 mapping rules with impairment effects under incompatible S1-R1 mapping rules. R1 were lateral key presses, and S2 were left/right pointing arrow heads. Both studies found the same pattern: when S1-R1 mapping was performed according to a simple, compatible mapping rule a substantial impairment effect was found, but the effect was absent when S1-R1 mapping required memorizing incompatible S1-R1 translations.

A definitive decision would require further research, in particular a more systematic investigation of the role of S1-R1 mapping rules in motorvisual priming.

### S2 modality in motor-perceptual priming

The motorvisual priming studies reviewed in this article were restricted to the visual domain. The ideomotor theory claims, however, that sensory effects in any modality can trigger actions. Previous action-perception studies have rarely applied other modalities (see Schütz-Bosbach and Prinz, 2007).

An interesting question for further research would be whether the temporal patterns found in motorvisual priming studies also obtain for motor-auditive or motor-tactile priming, or whether effect representations in different modalities are differentially involved in ideomotor cognition.

Another interesting issue is related to the interplay between different modalities. Research on multisensory interactions has shown that perceptual representations in one modality are tightly coupled to perceptions on other modalities when they frequently co-occur (Driver and Spence, 2000; Craig, 2006; Butz et al., 2010). Proponents of the ideomotor theory have often suggested that the perceptual representations involved in action selection are of a multisensory nature (e.g., Hommel, 2004).

This raises the question whether actions can also be *indirectly* triggered by, for example, auditory perceptual representation, when this representation is not a typical effect of the action but often co-occurs with its visual effects. This question could be answered by motor-perceptual priming. In particular, one would have to associate, in a learning phase, an action with an auditory effect that is compatible with a certain visual sensation, such as high-pitched tones with stimuli in the right visual field (see, e.g., Rusconi et al., 2006; Nishimura and Yokosawa, 2009; Eitan and Timmers, 2010). If this action, in a later dual task test phase, impaired perceptions in the other modality one could infer that ideomotor representations are multisensory.

### R1 type in motorvisual priming

The motorvisual priming studies reviewed in the present paper have been restricted to manual or verbal R1 responses, because these were predominant in the ideomotor-inspired literature on motorvisual effects.

A further well-researched motorvisual phenomenon is, however, the influence of eye movement planning on visual attention. Eye movements are very tightly coupled with vision, because they almost always have direct effects on visual input. It has long been known that the planning and execution of eye movements have a major impact on visual attention (Rizzolatti et al., 1987; Atabaki et al., 2009; Land and Tatler, 2009). The effects of eye movements on the perception of compatible stimuli can be facilitative (Shepherd et al., 1986; Sheliga et al., 1994; Hoffman and Subramaniam, 1995; Kowler et al., 1995; Smith et al., 2004), but also detrimental (Tibber et al., 2009). There is, however, already evidence from comparative studies that *eye* movements have qualitatively different effects on perception from *manual* movements (Fischer et al., 1999, 2003; Deubel and Schneider, 2003). It is an important task for future research to determine whether eye movements can be explained at least in part by ideomotor processes (see Herwig and Horstmann, 2011; Huestegge and Kreutzfeldt, 2012, for initial steps in this direction), and, if such an explanation is possible, why do ideomotor processes lead to different behavioral effects for eye and hand movements?

### Conclusion

Previous research on ideomotor processing has shown that action planning binds perceptual representations into a stable compound representation of the action. It is, however, still unclear which cognitive function this binding fulfills. Other open questions are the degree to which ideomotor representations are multisensory, and which types of actions employ ideomotor processing. These issues can potentially be solved by future motorvisual priming studies.

## Conflict of Interest Statement

The author declares that the research was conducted in the absence of any commercial or financial relationships that could be construed as a potential conflict of interest.
